# Suitability evaluation on material specifications and edible methods of Dendrobii Officinalis Caulis based on holistic polysaccharide marker

**DOI:** 10.1186/s13020-020-0300-7

**Published:** 2020-05-13

**Authors:** Zi-Jun Cao, Ka-Man Yip, Yi-Guo Jiang, Shi-Liang Ji, Jian-Qing Ruan, Cheng Wang, Hu-Biao Chen

**Affiliations:** 1 School of Chinese Medicine, Hong Kong Baptist University, Kowloon Tong, Hong Kong, China; 2 Department of Pharmacy, The Affiliated Suzhou Science and Technology Town Hospital of Nanjing Medical University, Suzhou, 215153 China; 3 Center for Drug Metabolism and Pharmacokinetics, College of Pharmaceutical Sciences, Soochow University, Suzhou, 215123 China

**Keywords:** Dendrobii Officinalis Caulis, Holistic polysaccharide marker (HPM), Commercial specification, Edible extraction methods, Immunomodulatory activity

## Abstract

**Background:**

Dendrobii Officinalis Caulis (DC) is a well-known tonic herbal medicine worldwide and has favorable immunomodulatory activity. Various material specifications of DC are available in herbal markets, and DC is ingested by different edible methods. However, whether these specifications and edible methods are suitable or not remains unknown.

**Methods:**

In this study, we evaluated the suitability of four material specifications (fresh stem, dried stem, *fengdou* and powder) and three edible methods (making tea, soup and medicinal liquor) based on holistic polysaccharide marker (HPM), the major polysaccharide components in DC. First, the HPMs were extracted from the four specifications of DC by the three edible methods in different conditions. Second, qualitative and quantitative characterization of the extracted HPMs was performed using high performance gel permeation chromatography (HPGPC). Third, immunomodulatory activities of the extracted HPMs were evaluated in vivo.

**Results:**

The results showed that the HPMs were found to be quantitatively different from various specification of DC and edible methods. In vivo analysis indicated that the HPMs exerted positive effects on innate immune responses by increment in proliferation of splenocytes, secretion of IL-2 and cytotoxicity activity of NK cells. Moreover, the dosage amount of HPM should be defined as a certain range, but not the larger the better, for exerting strong immunological activities.

**Conclusion:**

According to the both chemical and biological results, *fengdou* by boiling with water for 4 h is the most recommended specification and edible method for DC.

## Background

**Fig. 1:**
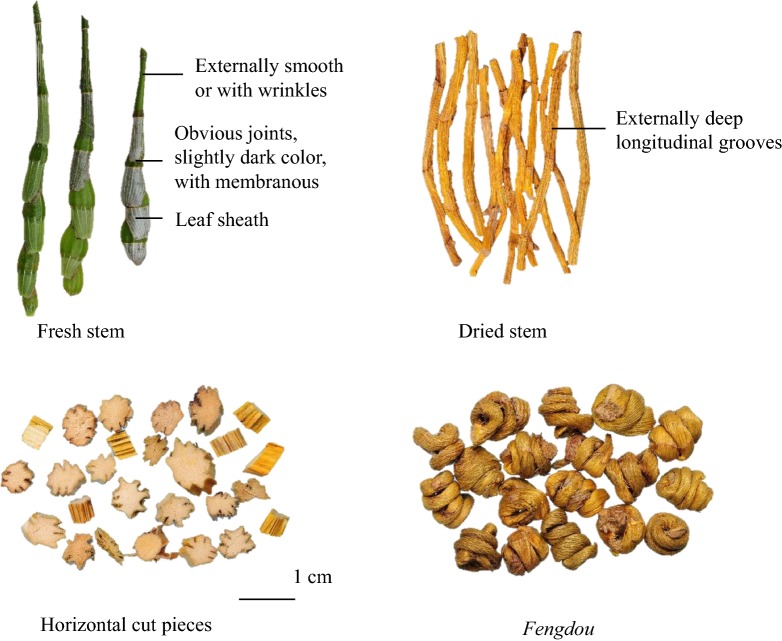
Different specifications of DC available in the herbal market

Dendrobii Officinalis Caulis (DC, *Tiepishihu* in Chinese) is the dried stem of *Dendrobium officinale* Kimura et Migo (Orchidaceae). As one of the best and well-accepted tonic traditional Chinese medicines, DC is not just consumed by patients, but also widely used in dietetic therapy or taken as daily supplements by the public. It could be easily found in herbal markets, drugstores and/or even supermarkets with various commercial specifications. For example, fresh or dried stem (sectioned stem, product name *Tiepishihu*, Fig. 1), *fengdou* (twisted dried stem, product name *Tiepifengdou*, Fig. 1) and powder are the typical specifications available in the market [1, 2]. These specifications of DC are frequently adopted with different edible methods, such as making tea, soup or medicinal liquor [3, 4]. However, these edible methods might not be the most suitable for DC consumption, i.e. the active ingredients in DC might not be fully obtained but retained in the dregs [5]. Previous research studies had also disclosed that quality and bioactivity of herbal medicines could be affected by commercial specifications [6, 7]. Regardless of this, scientifically supported recommendations on consumption methods of DC are still lacking, resulting in incomplete utilization and wastage of this valuable herb. Therefore, it is necessary to evaluate the feasibility and suitability of different specifications and edible methods of DC to provide guidance on the usage of DC.

With a unique chemical profile of saccharides up to 70% [8], polysaccharides are always regarded as an indicator for quality control of DC in official guidance, such as Pharmacopoeia of People’s Republic of China [9] and Hong Kong Chinese Materia Medica Standard [10]. Total sugar content determination of polysaccharides is the recorded quality control method for DC in the monographs of official guidance. It is usually achieved by colorimetric method [11], which is poor in specificity and thus inapplicable for qualitative characterization [12, 13]. Sugar composition analysis is another frequently employed analytical methods for quality control of polysaccharides [14]. It involves tedious procedures such as acid hydrolysis and derivatization, followed by chromatographic qualitative and quantitative determination of sugar derivatives. Due to its complicated operations, the results obtained from sugar composition analysis might be significantly varied [15–17]. Despite continuous efforts have been made for quality control of DC, these developed methods were far from satisfactory. Therefore, it is necessary to develop an efficient and reliable method for identifying and quantifying polysaccharides in DC. High-performance gel permeation chromatography (HPGPC) is a common size exclusion chromatography that separates polymers, such as polysaccharides, on the basis of molecular size [18]. It has been widely applied for qualitative authentication of polysaccharides. Purity and molecular weight determination can be obtained by characterizing peak symmetry and calculating with established retention time-molecular weight standard curve, respectively [19, 20]. Besides, based on our previous study, a HPGPC based strategy with improved macromolecular polysaccharides (> 60 kDa) retention and resolution was developed. With the developed method, a unique and specific polysaccharide peak was found in DC and used for simultaneous qualitative and quantitative analysis of polysaccharides [21]. The peak was accounted for over 90% of total polysaccharide contents in DC, so we separated it by ultra-filtration and deemed it as a holistic polysaccharide marker (HPM) for quality control of DC. This method is more efficient, stable and convenient in authentication and quality evaluation of DC, whereas it has been successfully applied for identification and quantification evaluation of DC in Hong Kong [22]. Therefore, the established specific and rapid HPGPC-based approach would be applied to evaluate the quality of DC with different specifications and consumption methods.

Pharmacological studies have demonstrated that polysaccharides did not just showed potential to serve as therapeutic tools [22] and health food [23]; but also possessed various biological activities, such as antioxidant [22, 24], immunological [25, 26] and anti-tumor activities [27]. In particular, polysaccharides were found largely associated with remarkable immune-modulating activities [28–31] of DC and other tonic Chinese herbal medicines [31–37]. Promotion of splenocytes proliferation, increase in cytokines secretions and enhancement of NK cell activities were commonly discovered in the related studies of immunomodulatory effects [38–41]. Therefore, besides chemical characterization, immunomodulatory effect is selected as the biological evaluation of differences in HPM.

Hence, in this project, we aim to investigate the immunomodulation effects of HPM from different specifications of DC and edible methods. First, HPM were qualitatively and quantitatively characterized by our developed method, in which their extraction time and yield were also tested. Next, the collected HPM were isolated, purified and biologically evaluated with regard to immunological activities in vivo. Lastly, summarized physicochemical and biological parameters of the HPM were compared to evaluate the specifications of DC and their edible methods, by which preferred specification and edible method were recommended to the public.

## Materials and methods

### Chemicals, reagents and materials

Ammonium acetate (purity ≥ 98%, chromatographic grade) was purchased from Sigma-Aldrich (St. Louis, MO, USA). Deionized water was prepared by Millipore Milli Q-Plus system (Bedford, MA, USA). HPM for chemical characterization was isolated in our laboratory as described previously [21].

Lentinan and YAC-1 cells were purchased from Tianjin Vientiane Hengyuan Technology Co., Ltd. (Tianjin, China). Phosphate-buffered saline (PBS), RPMI 1640 medium and penicillin–streptomycin (10,000 Unit/mL and 10,000 μg/mL) and were obtained from Gibco (Waltham, MA, USA) while fetal bovine serum (FBS) was obtained from HyClone (Logan, UT, USA). Potassium carbonate (KHCO_3_), ammonium chloride (NH_4_Cl) and ethylenediaminetetraacetic acid (EDTA), dimethyl sulfoxide (DMSO), and trypan blue solution (0.4%) were products of Sigma-Aldrich (St. Louis, MO, USA) while 3-(4,5-dimethylthiazol-2-yl)-2,5-diphenyltetrazolium bromide (MTT) was product of Invitrogen (Carlsbad, CA, USA). IL-2 ELISA kit was purchased from Shanghai ExCell Biology, Inc. (Shanghai, China) and LDH cytotoxicity assay kit was purchased from Thermo Fisher Scientific Inc. (Waltham, MA, USA).

Four different specifications of DC (fresh stem, dried stem, *fengdou* and powder) in three batches were obtained from the same supplier in local herbal market. They were collected from GAP base of *D. officinale* in Tiantai County, Zhejiang Province, China. Different specifications were prepared after collection of fresh stems of *D. officinale* at the GAP base. The stems were cut into sections of 10 cm long to obtain the specification of fresh stem (FS). Cut FS was dried at 50 °C for 24 h to obtain dried stem (DS). *Fengdou* (FD) was obtained from twisting FS into a spiral while heating, then dried at 50 °C for 24 h. While powder (P) was prepared by pulverizing DS to pass through 80-mesh sieve using a RT-04 grinder (Rong Tsong Precision Technology Co., Taichung, Taiwan). DC samples were authenticated by Prof. Chen Hubiao and voucher specimens of these samples were deposited at the School of Chinese Medicine, Hong Kong Baptist University, Kowloon Tong, Hong Kong.

### Preparation of HPM from different specifications by different edible methods

#### Determination of water contents for fresh stem

Water content in fresh stem was determined before extraction. It was conducted in duplicate according to the oven dried method stated in Volume IV of CP (2015 edition) and was calculated by the following equation:

$${\text{Water}}\;{\text{content}}\;(\% ) = {{{\text{Weight}}\;{\text{loss}}} \mathord{\left/ {\vphantom {{{\text{Weight}}\;{\text{loss}}} {{\text{Sample}}\;{\text{weight}}}}} \right. \kern-0pt} {{\text{Sample}}\;{\text{weight}}}} \times 100\%$$

#### Extraction procedure

Boiling with distilled water for 4 h (B), infusion with hot distilled water (W), and brewing with 45% ethanol (E) were the extraction methods for DC. For B, weighed samples were placed into a round-bottomed flask and extracted with heat under reflux for 4 h. For W, boiled distilled water (90 °C as initial temperature) was added to each conical flask containing weighed samples of different specifications. The mixtures were allowed to cool under room temperature and infuse for 4, 6 and 8 h, respectively. Similar procedures were applied for E but with 45% ethanol added to the samples and the brewing time were 168, 336 and 504 h, respectively under room temperature. All the extractions were conducted with sample to solvent ratio at 1:50 and in triplicate for each batch of specifications. The extraction methods for different specifications of DC were summarized in Table 1.

**Table 1:** Extraction methods for different specifications of DC

Specification	Method	Solvent	Temperature	Time
Fresh stems (FS)	1. Boiling 2. Infusion 3. Brewing	1. Distilled water 2. Distilled water 3. 45% ethanol	1. 100 °C 2. 90 °C as initial temperature (cool under room temperature) 3. Room temperature	1. 4 h 2. 4 h, 6 h and 8 h 3. 168 h, 336 h and 504 h
Dried stems (DS)				
*Fengdou* (FD)				
Powder (P)	1. Boiling 2. Infusion	1. Distilled water 2. Distilled water	1. 100 °C 2. 90 °C as initial temperature (cool under room temperature)	1. 4 h 2. 4 h, 6 h and 8 h

#### Isolation and purification of HPM

HPMs were isolated and purified from different DC extracts by our previous method [21]. In detail, each water extract (4 mL) was transferred into an ultra-centrifugal filter tube [molecular weight cut-off (MWCO) = 10 kDa] (Millipore, Billerica, MA, USA) and then centrifuged at 4000 rpm for 20 min, which the centrifugation was repeated for at least ten times. Finally, the separated HPMs retained on the filters were re-dissolved in 4 mL of distilled water and injected for qualitative and quantitative analysis.

For subsequent in vivo experiments, ethanol precipitation was applied for isolation and purification of the HPMs as it was more efficient for large-scale preparation of HPMs. The amount of ethanol required was optimized according to our previous study [42] and the finalized procedures were as follows. The extracts were precipitated by adding ethanol to make a final concentration of 62%, and left overnight at 4 °C. After centrifugation at 4000 rpm for 10 min, the precipitates were collected, washed with ethanol once, and dried (water bath, 70 °C) to remove any residual ethanol. Finally, the precipitates were completely re-dissolved in hot water (60 °C) and freeze-dried for further analysis.

#### Chemical characterization and content determination of HPMs

HPMs in different extracts were qualitatively and quantitatively analyzed using HPGPC performed on a Dionex UltiMate 3000 series ultra-high performance liquid chromatography and diode array detector (UHPLC-DAD) system coupled with Dionex Corona Veo charged aerosol detector (CAD) from Thermo Scientific (Waltham, MA, USA) was performed for the analysis. Two tandem TSK GMPWXL columns (300 × 7.8 mm i.d., 10 µm) were employed for analysis. Ammonium acetate aqueous solution (20 mM) was used as mobile phase at a flow rate of 0.6 mL/min. The column temperature was constantly kept at 40 °C. The parameters of CAD were set as follows: data collection rate at 2 Hz, filter at 10 s, gain at 100 pA, nebulizer heater at 60 °C and gas regulator mode at analytical. UV detection wavelengths were set at 260 and 280 nm. An aliquot of 20 µL solution was injected for analysis.

### In vivo immunomodulatory activity of HPMs from DC

#### Animals and cell lines

Institute of Cancer Research (ICR) male mice, 4 to 6 weeks old, 18–22 g, were obtained from Laboratory Animal Services Centre (LASEC) of The Chinese University of Hong Kong. The animals were housed in a temperature (24 ± 2 °C) and humidity (83 ± 2%) controlled environment with a 12 h light/dark cycle. Standard rodent chow and potable water were provided ad libitum during the whole experiment. All experiments with animals were carried out in accordance with the Animals Ordinance, Department of Health, Hong Kong Special Administration Region, PRC for the care and use of experimental animals. All of the experimental protocols were first approved by the Committee on Use of Human and Animal Subjects in Teaching and Research of the Hong Kong Baptist University. Before the experiment, the mice were acclimatized to the laboratory condition for 1 week.

ICR mice were randomly divided into control group (received distilled water), positive control group (treated with 100 mg/kg/day lentinan) and treatment groups (treated with low-, middle- and/or high-dose of HPMs obtained from different specifications and edible methods). The dosage of treatment groups were 0.9 g/kg/day, 1.35 g/kg/day and 1.8 g/kg/day for low-, middle- and high-dose of HPMs, respectively. The HPMs were given to the mice intragastrically for consecutive 14 days. On the 15th day, the mice were weighed and sacrificed through a cervical dislocation method.

YAC-1 cells were cultured in RPMI-1640 medium supplemented 10% fetal bovine serum (FBS) and 1% penicillin–streptomycin (complete medium) in a humidified atmosphere containing 5% CO_2_ at 37 °C.

#### Preparation of splenocyte suspensions

Modifications on the preparation for splenocyte suspensions in references [43, 44] were made and the detailed procedures were as follows. After scarification, spleens of ICR mice were collected aseptically and sliced into 2 fragments. One of the spleen fragments was stored with 1 mL PBS on ice for suspensions preparation while another was stored at − 80 °C for further analysis. To prepare the splenocyte suspensions, spleen fragments were pressed through a 40 µm mesh cell strainer (Biologix, Shandong, China) placed in a petri dish using the plunger end of a syringe. PBS was used to wash the cells through the strainers. The filtrates (total volume of 10 mL) were transferred into a 50-mL centrifuge tube and centrifuged at 1500 rpm for 10 min. The supernatants were removed, and the pellets were re-suspended in 3 mL of lysis buffer (0.15 M NH_4_Cl, 0.01 M KHCO_3_, and 0.1 mM EDTA, pH = 7.4) to lyse red blood cells. PBS (7 mL) was added to stop the reaction. After centrifugation at 1500 rpm for 10 min, the supernatants were discarded. 5 mL of RPMI 1640 medium were used to wash the pellet and removed after centrifugation. The cells were re-suspended in 10 mL of complete medium (RPMI 1640 medium supplemented with 10% FBS and 1% penicillin–streptomycin). Cell count and viability check of splenocytes were performed using trypan blue staining method with a hemocytometer. The cells were then adjusted to a concentration at 1.5 × 10^6^ cell/mL for further analysis [45].

#### Assay of splenocyte proliferation

The splenocyte proliferation was evaluated by MTT-based colorimetric assay as previously described with amendments [31, 46]. Briefly, 200 μL of each splenocyte suspensions at a concentration of 1.5 × 10^6^ cell/mL were incubated in a 96-well plate for 48 h (37 °C, 5% CO_2_). An equal volume of complete medium was used as vehicle control. 20 μL of MTT solution (5 mg/mL in PBS) were then added into the wells and incubated for another 4 h. After that, the 96-well plate was centrifuged at 1500 rpm for 10 min and the supernatants were discarded. DMSO (100 μL) was added to the precipitates to examine the proliferation of lymphocytes in splenocytes. The absorbance was determined at 570 nm in a Benchmark Plus microplate reader (Bio-Rad, Richmond, CA, USA) and the results were expressed as splenocyte proliferative index (SPI), which was calculated as ratio of optical density (OD) of treatment group to vehicle control cells:

$${\text{SPI}} = {{{\text{OD}}_{{{\text{treatment}}\;{\text{groups}}}} } \mathord{\left/ {\vphantom {{{\text{OD}}_{{{\text{treatment}}\;{\text{groups}}}} } {{\text{OD}}_{{{\text{vehichle}}\;{\text{control}}}} }}} \right. \kern-0pt} {{\text{OD}}_{{{\text{vehichle}}\;{\text{control}}}} }}$$

#### Secretion of IL-2 assay

Cytokine levels of spleen tissues were tested using commercial ELISA kits according to the manufacturer’s instructions. The homogenates of spleen tissues were processed according to reference with modifications [43]. To be specific, the thawed spleen fragments were weighed and prepared in 0.5 g/mL wet weight of 0.9% isotonic physiological saline. The spleen tissues were then homogenized with sonication for 30 s by using a sonifier (Branson, St. Louis, MO, USA). The samples were then centrifuged at 14,000 rpm for 10 min and the supernatants obtained were used to measure IL-2 levels according to the protocol of the assay kits.

#### NK cell cytotoxicity assay

NK cell activity was tested using the lactic acid dehydrogenase (LDH) method [28] according to the manufacturer’s instructions of the assay kits. The splenocytes were used as effector cells at 1.5 × 10^6^ cell/mL while YAC-1 cells were used as target cells at 3 × 10^4^ cell/mL to yield an effector/target cell ratio of 50:1. In the experimental groups, 50 μL of each effector and target cells were added per well into a 96-well plate. Cells in target maximum or spontaneous groups (50 μL) were treated with 50 μL complete medium giving a final volume of 100 μL. After incubation for 4 h (37 °C, 5% CO_2_), the NK cell activity was tested using LDH cytotoxicity assay kit and the following formula was used to calculate percentage of cytotoxicity.

$${\text{NK}}\;{\text{cell}}\;{\text{cytotoxicity}}\;(\% ) = {{\left( {{\text{OD}}_{\text{experimental}} - {\text{OD}}_{{{\text{effector}}\;{\text{spontaneous}}}} - {\text{OD}}_{{{\text{target}}\;{\text{spontaneous}}}} } \right)} \mathord{\left/ {\vphantom {{\left( {{\text{OD}}_{\text{experimental}} - {\text{OD}}_{{{\text{effector}}\;{\text{spontaneous}}}} - {\text{OD}}_{{{\text{target}}\;{\text{spontaneous}}}} } \right)} {\left( {{\text{OD}}_{{{\text{target}}\;{\text{maximum}}}} - {\text{OD}}_{{{\text{target}}\;{\text{spontaneous}}}} } \right)}}} \right. \kern-0pt} {\left( {{\text{OD}}_{{{\text{target}}\;{\text{maximum}}}} - {\text{OD}}_{{{\text{target}}\;{\text{spontaneous}}}} } \right)}} \times 100\%$$

### Statistical analysis

Content determination results of HPMs were expressed as mean ± standard error of mean (SEM). In vivo analysis results were expressed as mean ± standard deviation (SD). One-way analysis of variance (ANOVA) followed by the Fisher’s LSD tests using GraphPad Prism 6.0 (GraphPad Software, La Jolla, CA, USA) were applied to determine the differences between two compared data. The data with *p*-values less than 0.05 were considered statistically significant.

## Results

### Selection of extraction methods and times

In order to simulate the daily practice of preparing tea, soup and medicinal liquor of DC, extraction methods of infusion with hot water (W), boiling with water for 4 h (B) and brewing with 45% ethanol (E) were selected. It is known that long cooking time is required for preparing soup, i.e. Guangdong soup (*laohuotang* in Chinese) in daily life. It was also discovered that soup cooking within 4 h was safer than prolonged cooking time, for which the nitrite content unfavorable for health would be increased [47]. Therefore, the extraction time for B was set at 4 h. For W, it had been recorded that three to 4 h were needed for infusion of Chinese medicine [48], so 4 h of infusion was selected as the initial time. In order to examine the dissolvation rates of polysaccharides by this method, the extraction was conducted with 2-h interval until 8 h. For E, 45% ethanol was selected as the solvent since this concentration of ethanol was commonly used for wine-brewing [49]. The brewing time for medicinal liquor is usually 14 days (336 h) [50] while other suggested around 20 days [51]. Thus, 7 days were selected as the unit time for brewing and the period was set from 7 to 21 days (168 h to 504 h).

### Water content determination of FS

As water content in FS was so high that the HPM content comparison with other specifications would be affected, water contents of the three batches of FS were determined to decide the accurate amount of samples to be used. As shown in Table 2, the average water content of FS was 81.24%, for which suggested weighing 1.0 g were equivalent to about 0.2 g of samples. In order to have unbiased comparison of the HPM contents from different specifications and edible methods, instead of 1 g of samples used for the other specifications, 5.0 g of FS were used for extraction.

**Table 2:** Water contents of three batches of FS

Batches	Water content (%)^a^
FS 01	81.47
FS 02	83.52
FS 03	78.73
Mean	81.24
SD	2.40
RSD	2.96

^a^Average water content obtained from duplicate results

### Chemical characterization and content determination of HPMs

#### Typical chromatograms of different DC extracts

**Fig. 2:**
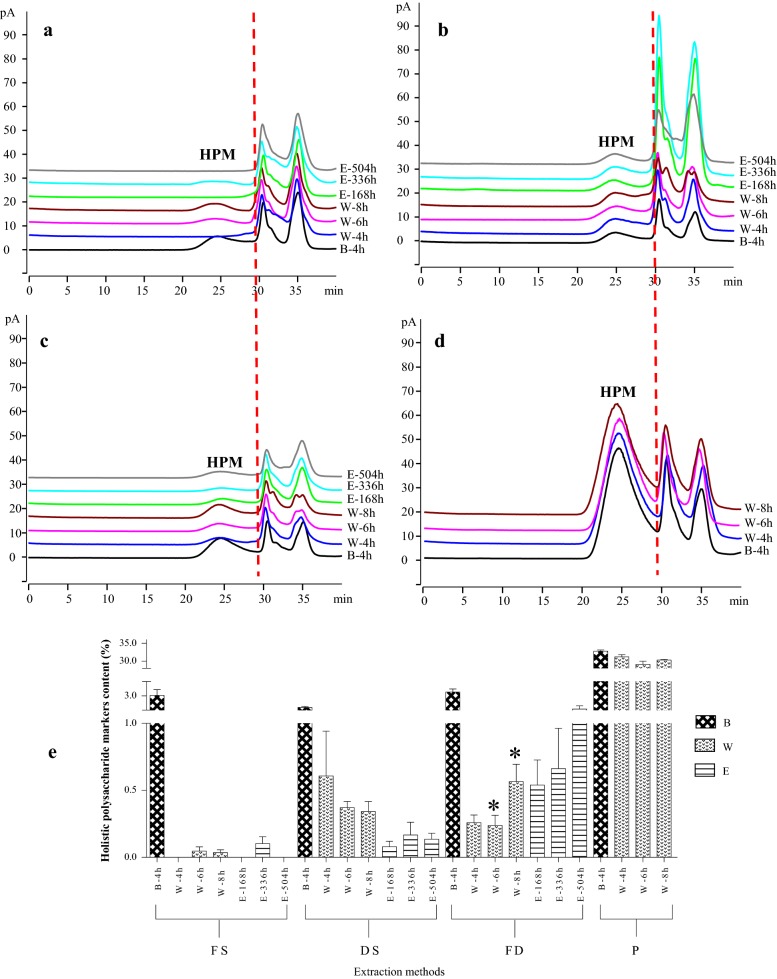
HPGPC-CAD chromatograms of DC extracts from **a** fresh stem, **b** dried stem, **c***fengdou* and **d** powder by different extraction methods and (E) content determination of HPMs in different DC extracts (*p < 0.05, compared with previous). *FS* fresh stem, *DS* dried stem, *FD fengdou*, *P* powder, *B* boiling, *W* infusion with hot distilled water, *E* brewing with 45% ethanol

The peaks showed no obvious absorbance under UV 260 nm and 280 nm (data not shown), suggesting the absence of free and conjugated nucleic acids or proteins and therefore no significant interference with polysaccharide analysis under the condition used. The typical HPGPC-CAD chromatograms of HPMs from different specifications and extraction methods were shown in Fig. 2a–d. The HPMs were consistently detected at 25.56 ± 1.48 min, which was in agreement with the previous study [21]. In Fig. 2a, none or small peak of HPMs was detected in FS with different extraction methods. DS (Fig. 2b) and FD (Fig. 2c) showed similar performances for HPM extraction while P (Fig. 2d) showed obvious HPM peak no matter the extraction method. The effects of extraction methods on obtaining HPMs were also shown in Fig. 2a–d. An obvious peak was found in the chromatograms of boiling with distilled water for 4 h (B-4 h) while relatively mere detections of HPMs were noticed with the other two extraction methods from different specifications (except P).

#### Effects of different specifications and extraction methods on amount of HPM obtained

To conduct the quantitative analysis as we previously demonstrated [21], HPM stock was isolated and purified from the DC extract of powder form with the extraction method of B-4 h as this combination obtained HPM the most. The freeze-dried HPM powder was used to prepare an aqueous stock solution (21.660 mg/mL). The stock solution was diluted to appropriate concentrations for construction of calibration curve. A series of appropriate concentrations of the solution in triplicate were injected into the HPGPC-CAD for analysis. Calibration curve was constructed by plotting logarithm of peak areas (y) against logarithm of HPM concentrations (x). The regression equation for the calibration curve was obtained as y = 0.8917x + 1.4093 (R^2^ = 0.9835), ranging from 0.001 to 21.660 mg/mL. Sample concentration was found between 0.004 and 7.117 mg/mL, which was included in the linear range for quantification.

The contents of HPMs from different DC specifications and extraction methods were determined by the established linear equation, and the results were shown in Fig. 2e. For FS, though brewing with 45% ethanol (E) and infusion with hot distilled water (W) did not yield high amount of HPM, the extraction times and content of HPM obtained were revealed. Effective dissolving-out time and the most suitable brewing time for HPMs in FS using E was 336 h (2 weeks). For W, no HPM was detected with the first 4 h of infusion but after the sixth hour and similar amount of HPMs were detected in both 6 h and 8 h (no significant difference was observed). Opposite phenomenon was observed in the specification of DS using W. HPM content was the highest at the fourth hour of infusion but a drop in the content as the infusion time increased to sixth hour was observed. For E with DS, similar trend was observed and the effective time for obtaining HPM was also 336 h. However, the specification of FD did not follow the comparable trend as FS and DS and showed significant change in HPM content by W (Fig. 2e). The content of HPM increased with the extraction times no matter with E or W and a significant increase in HPM content with eighth hour (*p* < 0.05) of infusion was observed. For P, HPM contents obtained were the highest, of which could be up to 30%, among different specification no matter using B or W.

### Selection of treatment groups for in vivo immunomodulatory analysis

**Fig. 3:**
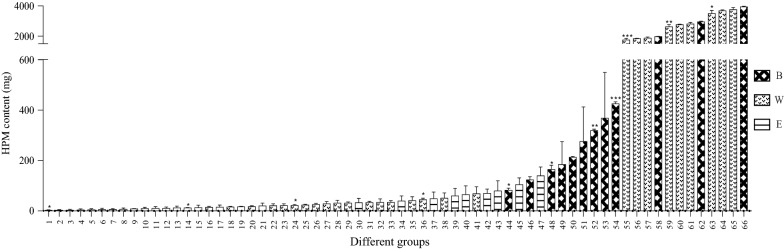
Statistical comparison of dosage amounts of HPMs obtained from different DC specifications by different extraction methods. (**1) FS W-8** **h(L)**; (2) FS W-6 h(L); (3) FS W-8 h(M); (4) FS W-6 h(M); (5) FS W-8 h(H); (6) FS W-6 h(H); (7) FS E-336 h(L); (8) DS E-168 h(L); (9) DS E-504(L); (10) FS E-336 h(M); (11) DS E-168 h(M); (12) DS E-336 h(L); (13) FS E-336 h(H); (**14) DS E-504** **h(M)**; (15) DS E-168 h(H); (16) FD W-6 h(L); (17) DS E-336 h(M); (18) FD W-4 h(L); (19) DS E-504 h(H); (20) DS W-4 h(L); (21) DS E-336 h(H); (22) DS W-8 h(L); (23) FD W-6 h(M); (**24) DS W-6** **h(L)**; (25) FD W-4 h(M); (26) DS W-4 h(M); (27) FD W-6 h(H); (28) DS W-8 h(M); (29) FD W-4 h(H); (30) FD E-168 h(L); (31) DS W-6 h(M); (32) FD W-8 h(L); (33) DS W-4 h(H); (34) FD E-336 h(L); (35) DS W-8 h(H); (**36) DS W-6** **h(H)**; (37) FD E-168 h(M); (38) FD W-8 h(M); (39) FD E-336 h(M); (40) FD E-168 h(H); (41) FD W-8 h(H); (42) FD E-504(L); (43) FD E-336(H); (**44) DS B-4** **h(L)**; (45) FD E-504(M); (46) DS B-4 h(M); (47) FD E-504(H); (**48) DS B-4** **h(H)**; (49) FS B-4 h(L); (50) FD B-4 h(L); (51) FS B-4 hM); (**52) FD B-4** **h(M)**; (53) FS B-4 h(H); (**54) FD B-4h(H)**; (**55) P W-6** **h(L)**; (56) P W-8 h(L); (57) P W-4 h(L); (58) P B-4 h(L); (**59) P W-6** **h(M)**; (60) P W-8 h(M); (61) P W-4 h(M); (62) P B-4 h(M); (**63) P W-6** **h(H)**; (64) P W-8 h(H); (65) P W-4 h(H); (66) P B-4 h(H). *FS* fresh stem, *DS* dried stem, *FD fengdou*, *P* powder, *B* boiling, *W* infusion with hot distilled water, *E* brewing with 45% ethanol, *L* low dose, *M* medium dose, *H* high dose (*p < 0.05; **p < 0.01; ***p < 0.001 compared with previous one)

For groups without HPM detection, they were excluded from the in vivo analysis. Low-, middle- and high-dose groups of HPMs were involved, for which the dosage amounts of HPMs were calculated according to the recommended intake of DC stated in CP 2015 (6–12 g) [9]. The dosage amount of all obtained markers was statistically compared, and the results were illustrated in Fig. 3. The low-, middle- and high-dose groups of obtained markers were rearranged with the dosage amount in ascending order. Then, the 1st group was respectively compared with the later ones (2nd, 3rd, 4th…) as far as significant difference (*p *< 0.05) was found, the group (i.e. 14th group) would be selected. Likewise, the selected group (the 14th group) was compared with the later ones (15th, 16th, 17th…) until significant differences were shown. Using this statistical method, 11 groups together with blank and positive control groups (totally 13 groups) were finally selected for immunological activities evaluation.

### In vivo immunomodulatory activity of HPM from DC

**Fig. 4:**
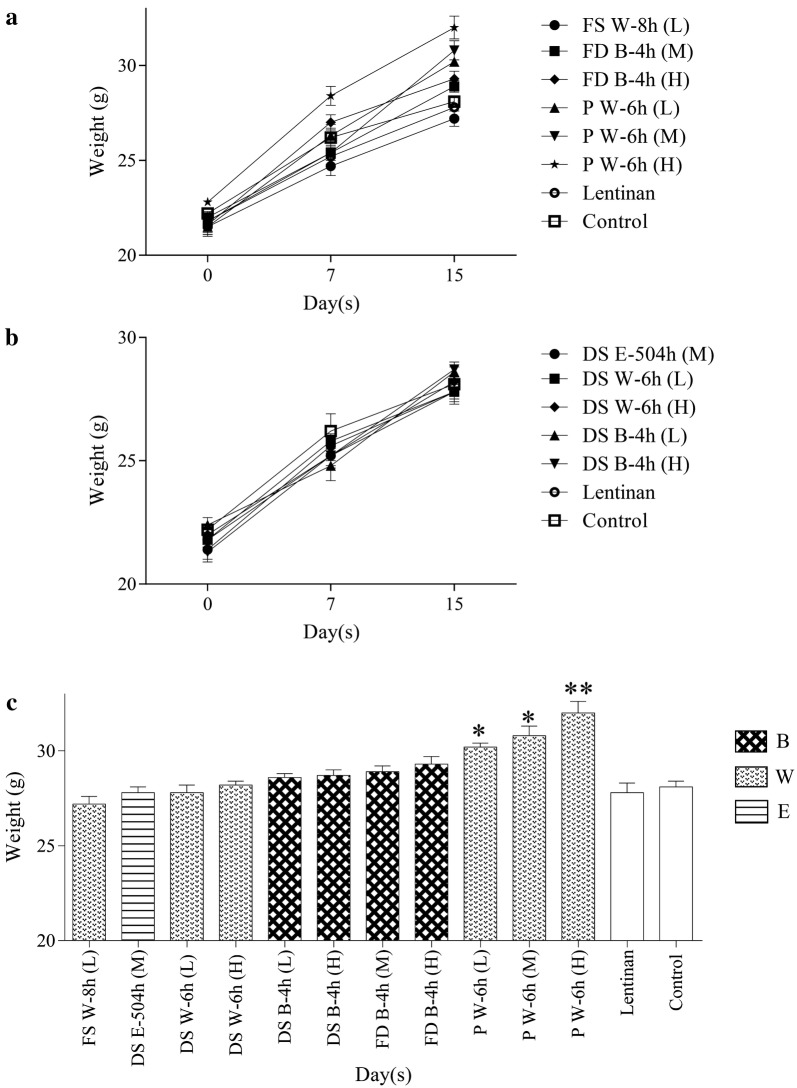
Growth curves (**a**, **b**) of mice in different groups and comparison of growth at day 15 (n = 6) (*p < 0.05, **p < 0.01, compared with control group). *FS* fresh stem, *DS* dried stem, *FD fengdou*, *P* powder, *B* boiling, *W* infusion with hot distilled water, *E* brewing with 45% ethanol, *L* low dose, *M* medium dose, *H* high dose

The effects of DC samples and lentinan (positive control) on growth of mice are shown in Fig. 4. There was a general increase in weight of mice after administration of DC polysaccharides and lentinan as shown in Fig. 4a and b. For the weight comparison on day 15 (Fig. 4c), it was observed that P W-6 h (L), P W-6 h (M), P W-6 h (H) could lead to significant weight gain (*p* < 0.05 and 0.01) as compared with control. However, this might not be representing the increase in immunomodulatory activity, further discussion on splenocyte proliferation, IL-2 production and NK cell cytotoxicity assay for immunity improvement by HPMs from different DC samples are shown below.

### Effects of HPMs on splenocyte proliferation

**Fig. 5:**
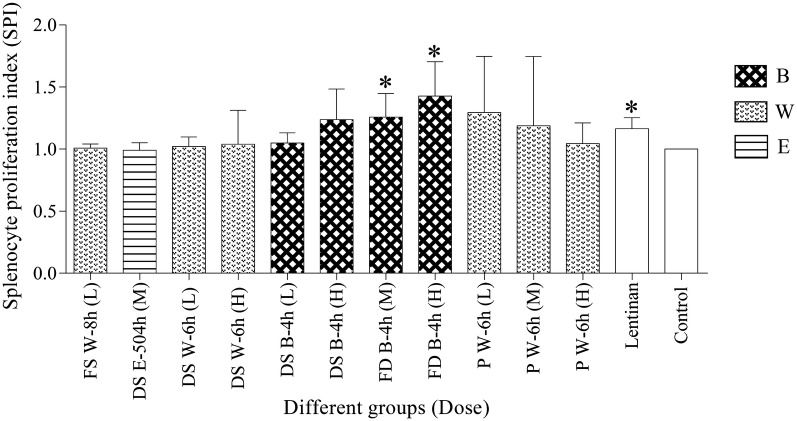
Comparison of SPI in different groups (n = 5) (*p < 0.05, compared with control group). *FS* fresh stem, *DS* dried stem, *FD fengdou*, *P* powder, *B* boiling, *W* infusion with hot distilled water, *E* brewing with 45% ethanol, *L* low dose, *M* medium dose, *H* high dose

As shown in Fig. 5 and Additional file 1: Table S1, the mice splenocytes were proliferated in all treated groups, among which FD B-4 h (M), FD B-4 h (L) and lentinan presented significant promotions as compared with control group (*p *< 0.05). The SPIs of 11 HPM groups increased with the dosage amount from FS W-8 h (L) to FD B-4 h (H), whereas it tended to decrease at the dosage amount of P W-6 h (L).

#### Effect of HPMs on secretion of IL-2

**Fig. 6:**
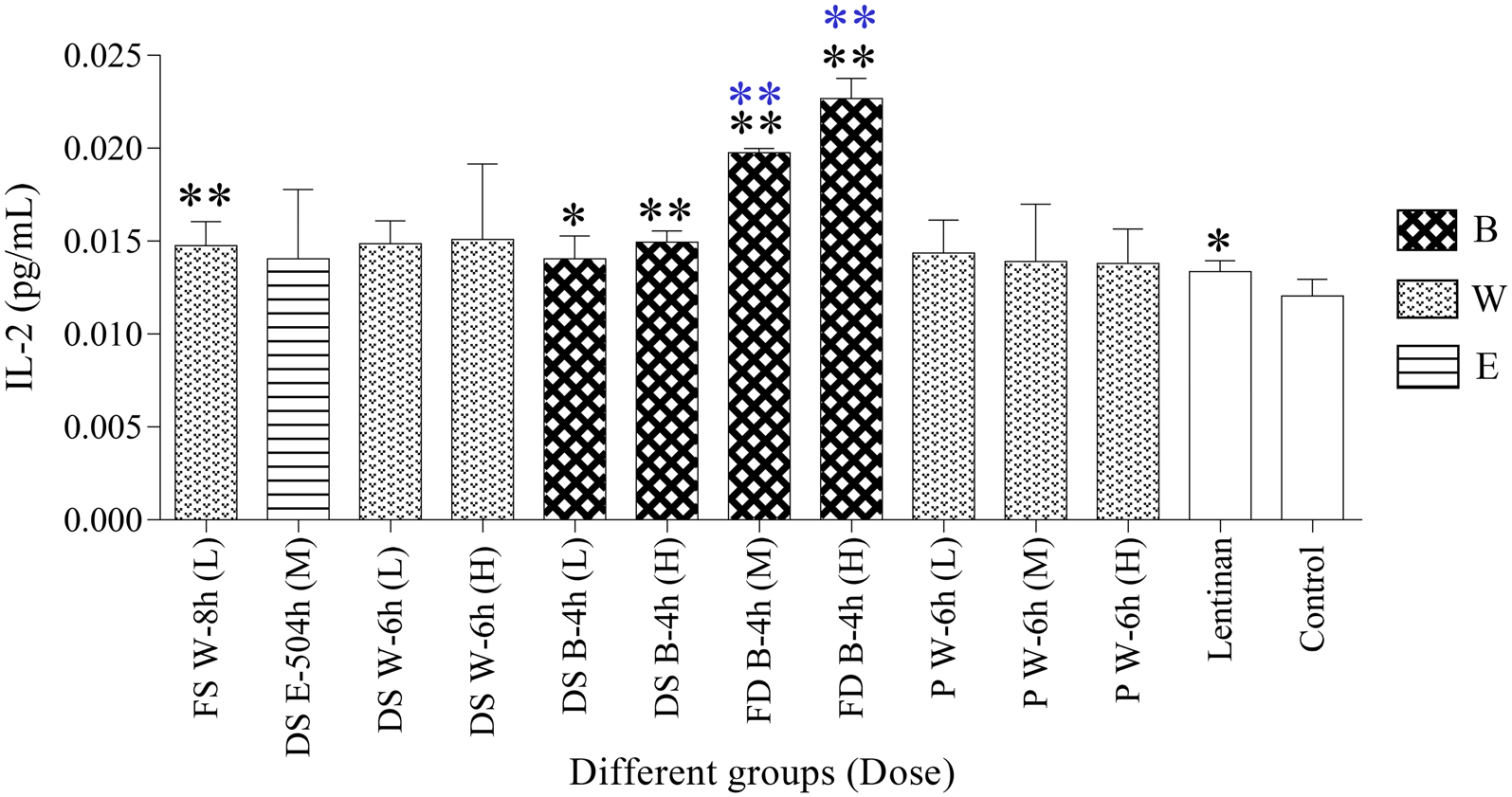
IL-2 content in different groups (n = 3) (*p < 0.05, **p < 0.01, compared with control group; **p < 0.01, compared with lentinan group). *FS* fresh stem, *DS* dried stem, *FD fengdou*, *P* powder, *B* boiling, *W* infusion with hot distilled water, *E* brewing with 45% ethanol, *L* low dose, *M* medium dose, *H* high dose

In the current study (Fig. 6; Additional file 1: Table S1), the secretion of IL-2 was increased to various degrees in the treated groups, among which five HPM-treated groups, including FS W-8 h (L), DS B-4 h (L), DS B-4 h (H), FD B-4 h (H) and FD B-4 h (M), together with the lentinan group showed a significant increase (*p *< 0.05 or *p *< 0.01) as compared with the control group. Moreover, the FD B-4 h (H) and FD B-4 h (M) groups even exerted better effects in secretion of IL-2 than the lentinan group (*p *< 0.01).

#### NK cell cytotoxicity activity assay

**Fig. 7:**
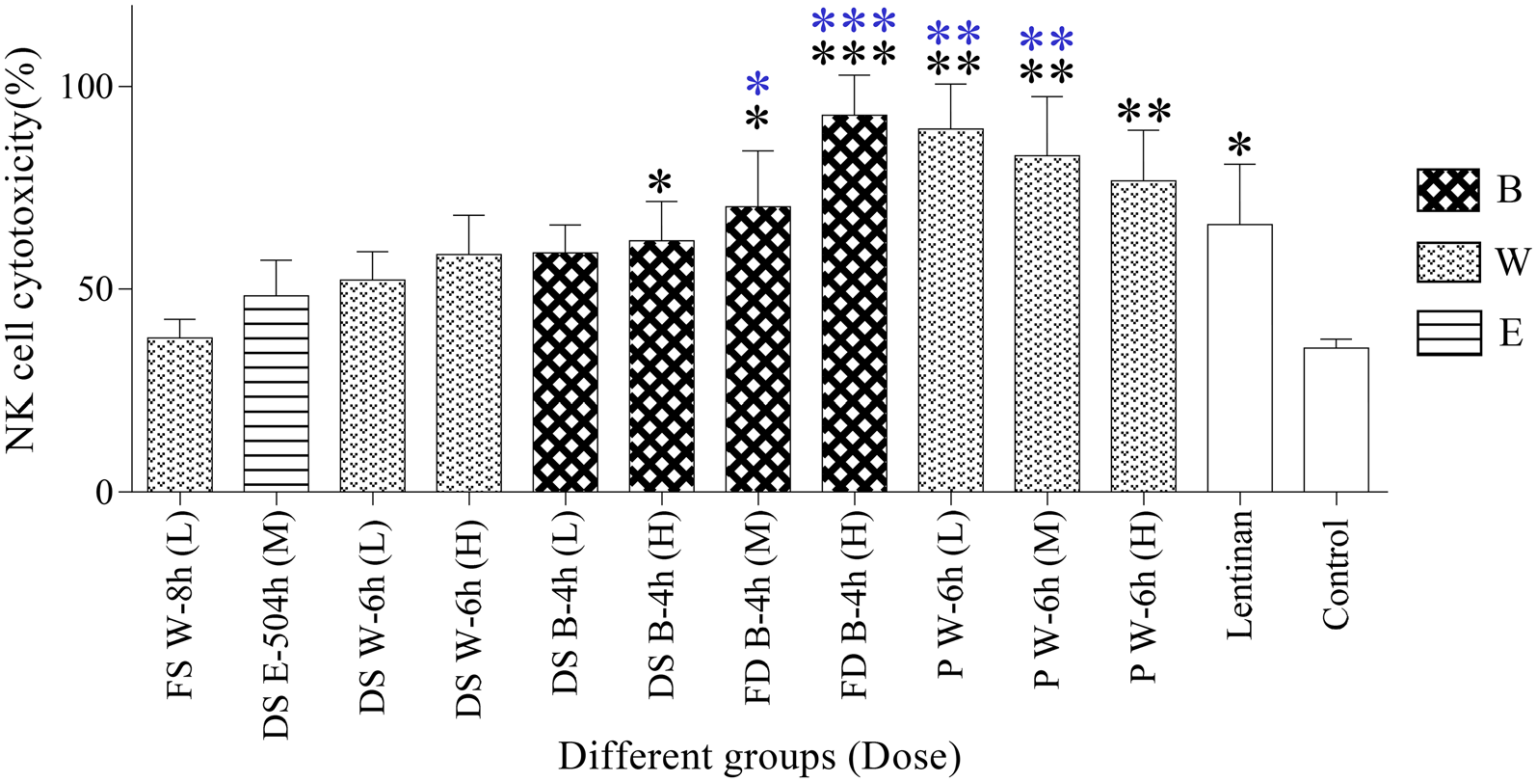
NK cell cytotoxicity activity in different groups (n = 4) (*p < 0.05, **p < 0.01, ***p < 0.001, compared with control group; *p < 0.05, **p < 0.01, ***p < 0.001, compared with lentinan group). *FS* fresh stem, *DS* dried stem, *FD fengdou*, *P* powder, *B* boiling, *W* infusion with hot distilled water, *E* brewing with 45% ethanol, *L* low dose, *M* medium dose, *H* high dose

As shown in Fig. 7 and Additional file 1: Table S1, the NK cell cytotoxicity activity was increased to different degrees in the treated groups, in which six polysaccharide-treated groups, including DS B-4 h (H), FD B-4 h (M), FD B-4 h (H), P W-6 h (L), P W-6 h (M), P W-6 h (H), and the lentinan group showed a significant increase (*p *< 0.05 or *p *< 0.01 or *p *< 0.001) when compared with the control group. Moreover, the FD B-4 h (M), FD B-4 h (H), P W-6 h (L) and P W-6 h (M) groups even exerted better effects in NK cell cytotoxicity activity than the lentinan group (*p *< 0.05 or *p *< 0.01 or *p *< 0.001); and FD B-4 h (H) group was found to be the most efficient for inducing NK cell activation.

## Discussion

HPM was characterized using HPGPC-CAD for chemical evaluation. The HPGPC-CAD chromatograms of different specification with different extraction methods are summarized in Fig. 2a–d and the corresponding quantification results are shown in Fig. 2e. From the results, several observations were noticed: (1) a trend of increase in the HPM content then decrease was observed for E with either FS or DS (Fig. 2a, 2b, e). The extraction time might be not enough for the first week (168 h) while 3 week-time (504 h) was too long as some micro-organisms might have degraded the HPM dissolved [52]. (2) At least 6 h were required for the extraction using W with FS (Fig. 2a, e). (3) It was not ‘as long as suitable’ for W with DS and 4 h of infusion was enough for HPM extraction (Fig. 2b, e). (4) Long extraction time were required for HPM extraction using W or E in the specification of FD (Fig. 2c, e). (5) For P, it was the most advantageous for HPM extraction as the contents of HPM were up to 30%. Based on Fig. 2 and these observations, it can be noticed that FS (Fig. 2a, e) was the least favorable commercial specification for HPM extraction, for which polysaccharides in FS could not be easily extracted. Obvious HPM peak and high content found with P (Fig. 2d, e) indicating this specification facilitated extraction of HPM the most despite the extraction methods used. While for extraction method, B-4 h was the most efficient despite the specifications used whereas W and E were comparable for HPM extraction (Fig. 2e). Although powder and boiling seemed to be the most effective specification and edible methods for DC, the conclusion could not be drawn without further biological analysis. Thus, HPMs from each extraction condition were prepared for the in vivo experiments to provide more evidences on selection of specification and edible methods.

It was known that polysaccharides from DC shown promising immuno-modulating effects [28–31], thus HPMs from different extracts were subjected to further in vivo analysis to discover if immune-enhancing effects were affected. Previous studies had shown that the immune-modulating effects of polysaccharides were dose-dependent [31, 46], while some revealed that high concentration was not necessary for strong stimulating effects [28]. This had led us concerning the importance of investigating the dose–effect relationship of HPMs from different specifications and extraction methods. Thus, low-, middle- and high-dose groups of HPMs were involved to examine the dose–effect relationship between polysaccharides and the immune-enhancing effects. However, in consideration of resource-saving and animal ethics, it was not preferred to compare the immunological activities of all obtained markers. 13 experimental groups (11 treatment groups, blank and positive control group) were used for immunological activities evaluation after statistical selection (Fig. 3). As DC is not just prescribed clinically, but also consumed as health food by the public, healthy mice were used for the in vivo experiments to see if its effects on immune system in physiological state. Lentinan was a polysaccharide studied extensively showing strong anti-cancer activity through activation of immune system of the host [53]. It was selected as the positive control since it was found to show immune-regulating effects no matter in healthy or pathologic animals [54] and had been subjected to clinical trials for cancer patients [55, 56].

As the body’s largest peripheral immune organ, spleen is the important place where lymphocytes are developed, and primary immune response takes places. By determining the status of spleen could acquire the information of immune functions [57]. The results showed that HPMs exerted significant immunomodulatory effects on innate immune responses mediated by spleen lymphocytes, which was in accord with previous studies of polysaccharides in DC [28, 31]. It had been noticed various polysaccharides from plant or animals improved spleen lymphocyte proliferation in dose-dependent manner [46, 58], but this result (Fig. 5; Additional file 1: Table S1) was consistent with other studies suggesting the opposite that a high amount might not be a must for effective immune-modulating effects [28, 59, 60].

Since cytokines play a prominent role in the development of immune responses [59], the effects of HPMs on cytokine secretion was also detected. Previous studies had proved that plant-derived polysaccharides could modulate the production of a range of cytokines from Th1 and Th2 cells in splenocytes [43, 58] and it had been found that Th1 cells were the main target of polysaccharides from DC [28, 31]. IL-2 is an important cytokine regulating the proliferation and differentiation of lymphocytes [61] and is mainly secreted by Th1 cells, thus, the change in IL-2 production was selected for the testing of the effects of different HPMs on immune system. Secretion of IL-2 (Fig. 6; Additional file 1: Table S1) exhibited similar trend with SPI (Fig. 5; Additional file 1: Table S1) showing a decrease at the dosage amount of P W-6 h (L), indicating that the effect of HPMs on the activity of IL-2 in spleen protein was also not in dose-dependent manner as previously mentioned [28, 59, 60].

As a member of the lymphocyte class, NK cells were best known for their nonspecific killing of tumor cells and virus-infected cells [62, 63]. There was evidence for NK cells in controlling infection in the earliest phases of the body’s immune responses, and hence NK cell activity assay has become a routine method for analysis of immune response and been used to test the anti-tumor activities of possible drugs [64]. It is known that increase of NK cell activity is induced by IFN-γ and IL-2 [65]; with the increase in IL-2 secretion (Fig. 6), the NK cell cytotoxicity activity shall also be increased. A similar trend of increase then decrease in NK cell cytotoxicity activity (Fig. 7; Additional file 1: Table S1) as SPI (Fig. 5; Additional file 1: Table S1) and IL-2 (Fig. 6; Additional file 1: Table S1) secretion was observed. This had again evidenced that a high dose was not essential for strong immuno-stimulating effects, which was in agreement with other researches on plant polysaccharides [28, 59, 60].

## Conclusion

In terms of chemical characterization, our research revealed that powder and boiling with water for 4 h were the best specification and edible extraction methods for HPM, respectively as highest yield of HPM was obtained. For the immunological activities evaluation, the results showed that HPMs from different specifications of DC using different extraction methods exerted positive effects on innate immune responses. Increment in spleen lymphocytes proliferation, IL-2 secretion and NK cells cytotoxicity were observed (Figs. 5, 6, 7 and Additional file 1: Table S1). The results had indicated that *fengdou* with boiling with water for 4 h gave the best immune-modulating effects. Moreover, we had revealed that the effect of the HPM on immune system was not dose dependent but within certain range of dosage amount. Therefore, for strong immunological activities of DC, *fengdou* (FD) by boiling with water for 4 h (B-4 h) is the most recommended specification and edible method for daily life consumption. As for other specifications of DC, the edible methods were suggested as follows: B-4 h for both fresh stem (FS) and dried stem (DS) and infusion with hot water for 6 h (W-6 h) using powder (P).

## Supplementary information

**Additional file 1: Table S1.** Mean and SD values of SPI index, IL-2 and NK cell cytotoxicity tests of different groups.

## Data Availability

The data sets used during the current study are available from the corresponding author on reasonable request.

## Abbreviations

DC: Dendrobii Officinalis Caulis
HPM: Holistic polysaccharide marker
FD: *fengdou*
FS: Fresh stem
DS: Dried stem
CP: Chinese Pharmacopoeia
LDH: Lactic acid dehydrogenase
