# Supplementary material for: Suitability evaluation on material specifications and edible methods of Dendrobii Officinalis Caulis based on holistic polysaccharide marker
Source: Chin Med. 2020 May 13;15:46. doi: 10.1186/s13020-020-0300-7 (PMC7218507; doi:10.1186/s13020-020-0300-7)
Supplement: Supplementary file 1 — Additional file 1: Table S1. Mean and SD values of SPI index, IL-2 and NK cell cytotoxicity tests of different groups. [file 13020_2020_300_MOESM1_ESM.docx]

**Additional file 1: Table S1.** Mean and SD values of SPI index, IL-2 and NK cell cytotoxicity tests of different groups.

| **Groups** | **SPI Index** | **IL-2** | **NK cell cytotoxicity** |
| --- | --- | --- | --- |
|  | Mean ± SD | Mean ± SD  (pg/mg) | Mean ± SD  (%) |
| Fs W-8h (L) | 1.007 ± 0.03 | 0.0148 ± 0.0013 | 37.9.5 ± 4.61 |
| Ds E-504h (M) | 0.991 ± 0.06 | 0.0141 ± 0.0037 | 48.336 ± 8.78 |
| Ds W-6h (L) | 1.021 ± 0.08 | 0.0149 ± 0.0012 | 52.249 ± 7.00 |
| Ds W-6h (H) | 1.040 ± 0.27 | 0.0151 ± 0.0041 | 58.547 ± 9.74 |
| Ds B-4h (L) | 1.050 ± 0.08 | 0.0140 ± 0.0012 | 58.956 ± 6.88 |
| Ds B-4h (H) | 1.237 ± 0.25 | 0.0150 ± 0.0006 | 61.911 ± 9.78 |
| Fd B-4h (M) | 1.258 ± 0.19 | 0.0198 ± 0.0002 | 70.310 ± 13.81 |
| Fd B-4h (H) | 1.428 ± 0.28 | 0.0227 ± 0.0011 | 92.970 ± 9.92 |
| P W-6h (L) | 1.294 ± 0.45 | 0.0144 ± 0.0018 | 89.528 ± 11.15 |
| P W-6h (M) | 1.188 ± 0.56 | 0.0139 ± 0.0031 | 82.917 ± 14.62 |
| P W-6h (H) | 1.044 ± 0.17 | 0.0138 ± 0.0018 | 76.706 ± 12.58 |
| Lentinan | 1.164 ± 0.09 | 0.0134 ± 0.0006 | 65.950 ± 14.87 |
| Control | 1.000 ± 0.00 | 0.0120 ± 0.0009 | 35.429 ± 2.24 |
